# Comparative management practices of Wilson disease in Californian and Italian providers

**DOI:** 10.1186/s41043-025-01072-1

**Published:** 2025-09-30

**Authors:** Sandy Vien, Colin McNamara, Ronald Hsu, Massimo Zuin, Valentina Medici

**Affiliations:** 1 Department of Internal Medicine, University of California Davis, 4150 V street, California 95817 Sacramento, USA; 2 Department of Internal Medicine, Division of Gastroenterology and Hepatology, University of California Davis, 4150 V street, Sacramento, California 95817 USA; 3 UO Medicina Generale Epatologia e Gastroenterologia Medica ASST Santi Paolo e Carlo, Milano, Italy

**Keywords:** Copper, Survey, Italy, California, Wilson disease, Diagnosis, Treatment

## Abstract

**Background:**

There is a scarcity of randomized and high-quality studies to aid clinicians in management and treatment of Wilson disease (WD). Even amongst society practice guidelines in North America and Europe, diagnosis and management of WD varies. The aim of this study is to elucidate WD diagnosis and treatment patterns by conducting a survey of clinicians in California and comparing the results to clinicians in Italy as a representation of European practices.

**Methods:**

We developed a 51-item survey assessing WD diagnostics, therapeutics, and disease monitoring. The survey was distributed through email to 1330 California gastroenterologists, hepatologists, and movement neurologists and to multiple Italian academic medical centers.

**Results:**

Thirty-two providers in California completed the survey encompassing a total of 236 patients. Twenty-three providers in Italy with a total of 390 patients in their care responded. About half of California providers perform a full neurologic evaluation before initiating therapy in patients with predominantly hepatic presentation while 71% of Italian providers perform one. In patients with predominantly hepatic presentation, 47.4% of California providers use trientine as initial therapy, 26.3% use d-penicillamine, and 10.5% use combination therapy with chelators and zinc. No one reported using zinc monotherapy as initial treatment. Italian providers report using d-penicillamine as initial therapy in 85% of cases, followed by zinc salt (10%), and none uses trientine. WD patients on combination therapy with chelators and zinc are followed by 34% of California respondents and 32% of Italian respondents. In patients with predominantly neurologic manifestations, initial therapy choices are variable with 38.9% of California providers using d-penicillamine, 16.7% using zinc salts, 11.1% using trientine, and 22% using other therapies. 55% of Italian providers use d-penicillamine, 20% combination chelator and zinc, 15%, trientine and 10% zinc salts. Changing from initial therapy to maintenance therapy in both surveys occur after stabilization of clinical presentation, liver function tests, and 24-hour urinary copper in 72% and 86% of California and Italian providers respectively.

**Conclusions:**

Our findings highlight the significant variability in initial therapies for WD amongst California and European/Italian providers. Despite the wide use of combination therapy of chelators and zinc, its needs further exploration.

**Supplementary Information:**

The online version contains supplementary material available at 10.1186/s41043-025-01072-1.

## Background

Wilson disease (WD) is a rare, autosomal recessive disorder due to excessive copper accumulation mainly in the liver and the brain due to mutations in *ATP7B* gene [1]. The prevalence of the disease is generally believed to be 1/30,000 to 1/100,000 individuals with a carrier frequency of 1/90. However, more recent studies obtained from next generation sequencing suggest that the genetic prevalence is closer to 1/7000 [2, 3]. A majority of the new diagnoses affects children and young adults, although there are cases that present in late adulthood [4]. The clinical manifestations involve hepatic disease as well as neurologic and neuropsychiatric signs and symptoms, with typical ophthalmic findings being Kayser-Fleischer rings [2]. Severity of symptoms varies in presentation including elevation in liver enzymes to chronic hepatitis and cirrhosis or fulminant liver failure. Treatment of WD is lifelong with the use of pharmacotherapy through chelating agents to promote urinary copper excretion or blockage of copper absorption and dietary copper restriction [5]. Liver transplantation is considered for severe cases and those refractory to pharmacotherapy. If left untreated, outcomes can be fatal. Early diagnosis of WD is essential to initiation of treatment to prevent disease progression, as cirrhosis at the time of diagnosis has been associated with the worst outcomes [6]. However, diagnosis is often delayed with the mean time between appearance of symptoms and diagnosis often exceeding two years [7]. Lack of provider awareness to rare and atypical clinical presentations of WD may contribute to this lapse.

Currently, there is a scarcity of randomized and high-quality studies to aid clinicians in management and treatment of WD [8]. In addition, the management of WD is challenged by the need of monitoring multiple parameters of copper homeostasis, including 24-hour urinary copper and non-ceruloplasmin copper which is difficult to quantify and interpret. Several survey studies described clinician experiences with diagnosis and treatment of WD patients and patient outcomes [9–12]. Presently, there are three society guidelines/guidances providing recommendations for management of WD including the recommendations from American Association for the Study of Liver Disease (AASLD), European Association for the Study of the Liver (EASL), and European Society for Pediatric Gastroenterology, Hepatology and Nutrition (ESPGHAN) [5, 13, 14]. Even between guidelines, there are differences in recommendations across all aspects of patient care. One example would be different diagnostic approaches for suspected WD cases with AASLD guidelines using an algorithm including biochemical markers with diagnostic scoring systems as complementary tools versus guidelines from EASL and ESPGHAN which favor the utilization of the Leipzig scoring system. Moreover, clinicians do not always follow the published guidelines. One study from the US showed that only 40% reported doing so while another study reported 41% of European clinicians following guidelines [12, 15]. A real-world retrospective study of 225 patients showed lack of consensus in the US regarding first-line therapy for WD treatment [16]. It is imperative for additional studies to be conducted to further aid clinicians in the management of WD as patients are affected by higher mortality rates, longer length of stays while hospitalized and increased hospitalization costs [17]. Because the clinical management of WD is varied, we wish to further elucidate practice patterns of clinicians by adopting a novel approach based on a comparative survey between clinicians in California and Italy as California has one of the highest health-insured population in the US and Italy has a universal healthcare system [18, 19]. Of note, this is the first study proposing a comparison between two regions. Each represents different WD society guidelines, with California having two Centers of Excellence for WD whereas Italy has major WD referral centers.

## Methods

A cross-sectional survey was developed by M.Z. and V.M. and conducted to assess the current practices of WD management in California and Italy. In January 2022, the survey was distributed via email to 23 referral centers in Italy (Supplemental Table 1). A similar survey was sent to 1,330 California gastroenterologists, hepatologists, and movement neurologists at major academic centers in January 2024. The survey was based on 51 questions (Supplemental Table 2), including multiple choice questions and open-ended questions (Supplemental material, Appendix 1). Some questions were modified to adjust the language from Italian universal health care to US insurance-based coverage. The study was approved by UC Davis Institutional Review Board and surveyed physicians (participants) provided their consent to participate to the study (protocol #2022217-1).

Of note, AASLD guidance for the management of WD were published in April 2023, shortly before this survey was distributed to California providers and EASL-ERN Guidelines were published in April 2025, after the survey was distributed to Italian centers.

## Results

Twenty-three surveys were returned from Italian providers, caring for 390 WD patients with 61% following adult patients. Hepatologists comprised of 52.2% of responses, pediatrics 30.4%, neurologists 8.7%, and internists 8.7%. Thirty-two surveys (2.4%) were completed by California physicians caring for 236 WD patients with 98% following adult patients. 48% of California providers report practicing in referral centers for rare diseases, in comparison to 95% of Italian providers. 56% of California respondents say that WD patients are followed by a single dedicated specialist versus 95% of Italian respondents. About 60% of both California and Italian providers report that WD patients at their center are followed by multidisciplinary teams. 60% of California providers diagnosed 1–5 patients with WD in their professional life, compared to 35% of Italian providers.

### Diagnostics

68% of California providers and 52% of Italian providers state that their center performs genetic analysis for WD, receiving results mostly within 1 month. Majority of centers in both surveys have access to laboratories that perform 24-hour quantification of urinary copper and zinc (91% in California, 76% in Italy). For cases with primarily hepatic presentation with no neurological symptoms, 51% of California providers always obtain a full neurologic evaluation and/or brain MRI before therapy compared to 71% of Italian providers. An eye exam to evaluate for Kayser-Fleischer rings is completed by 81% of California respondents and 95% of Italian respondents. At the time of diagnosis of WD, 50% of California providers report obtaining a liver biopsy with copper quantification in all cases, in contrast to 28% of Italian providers. About 40% of Californian and 48% of Italian providers obtain a liver biopsy with copper quantification for diagnostic purposes only in select cases.

### Therapeutics and monitoring

**Fig. 1:**
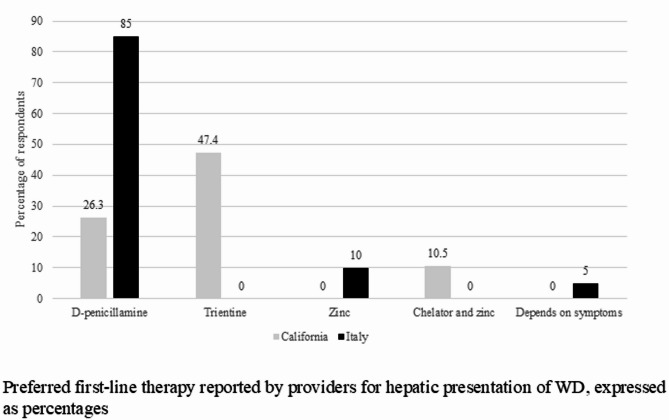
Preferred first-line therapy reported by providers for hepatic presentation of Wilson disease (WD), expressed as percentages of respondents. 85% of Italian respondents versus 26.3% of California respondents report choosing d-penicillamine as initial therapy for hepatic presentations of WD. 47.4% of California providers chose trientine as initial therapy

**Fig. 2:**
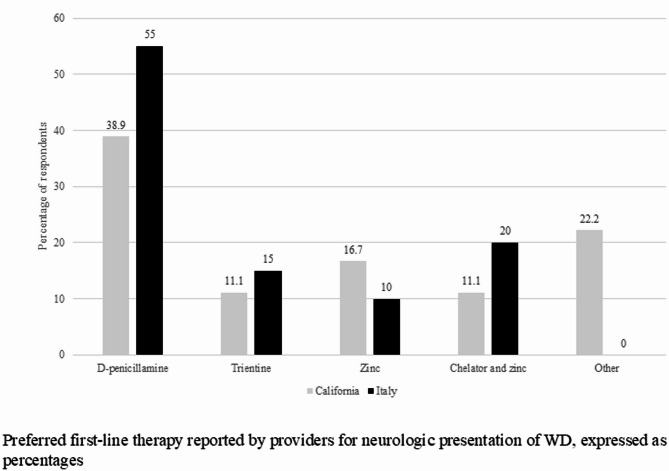
Preferred first-line therapy reported by providers for neurologic presentation of Wilson disease, expressed as percentages of respondents. Initial chosen therapies for neurologic presentations by both California and Italian respondents were variable

**Fig. 3:**
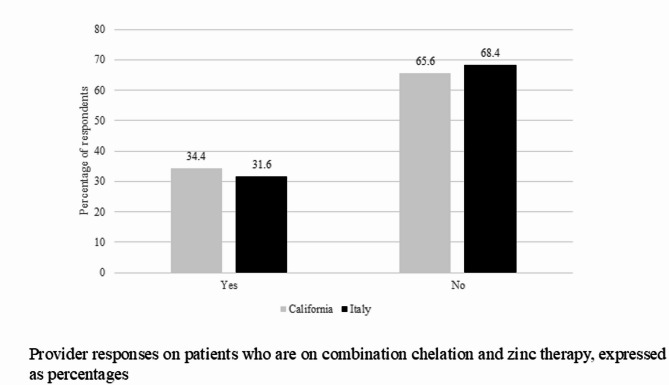
Provider responses on patients who are on combination chelation and zinc therapy, expressed as percentages of respondents. A significant number of patients on combination chelation and zinc therapy were reported by both California and Italian providers

Italian providers opt for d-penicillamine as first choice in most of the cases both for hepatic and neurological presentations and none claim to recommend trientine. In patients with predominantly hepatic presentation, 47.4% of California providers use trientine as initial therapy, followed by d-penicillamine, and combination therapy with association of chelators and zinc (Fig. 1). None o of the surveyed provider reported using zinc monotherapy as initial treatment. In patients with predominantly neurologic manifestations, initial therapy choices are variable with 38.9% of California providers using d-penicillamine, 16.7% using zinc salts, 11.1% using trientine (Fig. 2). 55% of Italian providers use d-penicillamine, 20% combination chelator and zinc, 15% trientine and 10% zinc salts in patients with neurologic symptoms. WD patients on combination therapy with chelators and zinc are followed both by California (34%) and Italian (32%) respondents (Fig. 3). Over 90% of California providers say that they choose initial therapy based off guidelines versus 73% of Italian providers. Changing from initial therapy to maintenance therapy in both surveys occur after stabilization of clinical presentation, liver enzyme levels, and 24-hour urinary copper in more than 50% of both groups of providers. During the maintenance phase of WD, 73% of California providers and 47% of Italian providers order blood tests every 6 months to monitor the patients.

## Discussion

In this cross-sectional study conducted in two countries with universal or high rates of access to healthcare, there were several major findings relevant to WD management. Firstly, 48% of California providers practiced in referral centers for rare diseases whereas 95% of Italian respondents were at major referral centers. Consequently, 56% of California providers have WD patients followed by a single dedicated expert versus 95% of Italian providers. This indicates that care of WD patients was more fragmented among various providers in California, compared to Italy where more WD care was provided in major referral centers. Secondly, in patients with primarily hepatic presentations, 51% of California providers obtain a full neurologic evaluation and/or brain MRI before therapy compared to 71% of Italian providers. Thirdly, for WD patients with predominantly hepatic presentation, nearly half of California providers report prescribing trientine as first-line therapy vs. 85% of Italian providers who report choosing d-penicillamine. Fourthly, our study shows that there is large variability in provider choices for initial therapy in patients with predominantly neurologic symptoms possibly due to unclear society guidelines. Lastly, the usage of combination therapy with chelators and zinc is reported in both cohorts of respondents, despite limited data in its utility.

It is well known that patients with rare diseases such as WD have greater unmet needs with delayed diagnosis being often complicated by progressive, irreversible sequelae of the disease with limited treatment options [6, 20]. Timely referrals to referral centers may aid in earlier diagnosis and likely to be associated with improved outcomes due to interdisciplinary collaborations [21]. Noting these differences in distribution of care for WD between California and Italy, further studies are needed to understand if there are differences also in patient outcomes. Only on the most recent EASL society guidelines, it has been established that all WD patients should have a neurologic assessment [13], thus surveyed providers adopted various approaches and likely will continue to adopt various approaches until the new guidelines become established practice. One retrospective study of 48 children with neurologically symptomatic and asymptomatic WD showed brain magnetic resonance imaging (MRI) changes may occur in hepatic WD, regardless of the presence of neurologic symptoms and recommended brain MRI prior to initiation of treatment [22]. Per AASLD WD guidances, in patients with neurologic signs or symptoms, referral to a neurologist or movement disorder specialist to perform a comprehensive neurologic examination is recommended. However, there are limited recommendations on those without neurologic manifestations.^5^ The 2025 EASL guidelines clearly state that all adults diagnosed with WD should receive a detailed neurological exam. However, this recommendation was not stated in the 2012 guidelines [23]. Obtaining imaging, and in particular brain MRI should complete the assessment of WD, though serial imaging has limited utility in determining prognosis or monitoring neurologic progression [5]. 

In all society guidelines, the recommended initial treatment for symptomatic WD is copper chelation, with d-penicillamine or trientine. ^4,5,14^ [4, 5, 14] D-penicillamine and trientine have comparable outcomes as effective therapies for WD, though d-penicillamine is likely to have higher rates of adverse effects [24]. Additional studies have shown that trientine is effective and well tolerated in patients who have withdrawn from d-penicillamine [25]. In the CHELATE trial, an open-label, noninferiority phase 3 trial comparing d-penicillamine and trientine tetrahydrochloride, the latter was found to be non-inferior to d-penicillamine [26]. There is a significant difference with initial therapy choices between California and Italian providers. Differences in initial therapy choices may be due to multiple factors including concerns on drug side effect profiles, drug availability, and costs [24, 27]. In addition, trientine tetrahydrochloride has been approved for patients intolerant of d-penicillamine in Europe since 2017 and since 2022 in the US [28]. 

Chelation therapy with d-penicillamine or trientine is recommended for symptomatic patients, however the potential for neurologic worsening does need to be considered in choosing initial treatment [29]. With the currently available chelating agents, it is unclear as to whether one has higher potential for neurologic deterioration. Some centers suggest that chelation therapy should be reduced or stopped if neurologic worsening occurs and zinc therapy should be used instead [29]. 

A large proportion of WD patients both in California and Italy (34%, 32% respectively) appear to receive a combination therapy with chelators and zinc. Small case reports and series suggest possible benefits in using combination therapy for severe liver disease due to different mechanisms of action with zinc and oral chelators [30–32]. However, a large systematic review concluded no significant effectiveness of combination therapy compared to monotherapy and higher mortality rates in the combination therapy cohort compared to those using other medications [33]. Furthermore, the practicality of administering drug doses may not be ideal for medication adherence as oral chelators and zinc must be taken at different times for optimal bioavailability [34]. The superiority of combination therapy over monotherapy remains unproven.

The strengths of the present work include the large number of WD patients followed by practicing physicians in both countries and the comparison between two regions. Despite these strengths, our study has a few limitations. The response rate of California providers was very low, especially in comparison with the European counterpart. This raises the possibility of sampling bias and in general of poor representation of California practice, although California providers appear to follow a large number of WD patients. The survey is solely based on the recall of the physicians who may not accurately remember past events. Additionally, there may be self-report bias that is not reflective of actual management of WD patients as majority of providers reported following WD society guidelines, but prior studies have shown that may not be the case [15]. Another limitation is that some specialists may be following the same patients providing overlapping data. Regardless, our study provides a unique insight into WD management in different countries/regions characterized by high access to high quality health care and still with various interpretations of society guidelines and guidances.

## Supplementary Information

Below is the link to the electronic supplementary material.

Supplementary Material 1

## Data Availability

All data generated or analyzed during this study are included in this published article and in Supplemental Table 1.
