# Supplementary material for: Comparative management practices of Wilson disease in Californian and Italian providers
Source: J Health Popul Nutr. 2025 Sep 30;44:339. doi: 10.1186/s41043-025-01072-1 (PMC12487098; doi:10.1186/s41043-025-01072-1)
Supplement: Supplementary file 1 — Supplementary Material 1 [file 41043_2025_1072_MOESM1_ESM.docx]

**SUPPLEMENTAL MATERIAL**

**Supplemental Table 1.**

| **Survey question** | **Italy respondent results** |
| --- | --- |
| 1. What type of patients do you follow? | Adult: 73.9%; Pediatric: 47.8% |
| 1. Do you practice within a referral center for rare disease? | Yes: 95.7%; No: 4.3% |
| 1. Approximately how many adult patients with Wilson disease are followed in your center? | Variable |
| 1. How many patients have you diagnosed with Wilson disease in your professional life? | 0 patients: 8.7%; 1-5: 34.8%; 6-10: 13%; 11-20: 21.7%; over 20: 21.7% |
| 1. How many patients have been diagnosed with Wilson disease in the past three years? | 0 patients: 17.4%; 1-5: 65.2%; over 5: 17.4% |
| 1. How many of your patients are being treated with d-penicillamine therapy? | 0 patients: 5.3%; 1: 10.5%; 2: 5.3%; 3: 10.5%; 4: 31.6%; 5: 5.3%; 6: 5.3%; 7: 5.3%; 8: 5.3%; 22: 5.3%; 40: 5.3%; 60: 5.3% |
| 1. How many of your patients are being treated with trientine dihydrochloride therapy? | 0 patients: 84.2%; 1: 10.5%; 3: 5.3% |
| 1. How many of your patients are being treated with trientine tetrahydrochloride therapy? | 0 patients: 50%; 1: 15%; 2: 10%; 3: 5%; 5-7: 5%; 8: 5%; 18: 5%; 45: 5% |
| 1. How many of your patients are being treated with zinc salts? | 0 patients: 21.1%; 1: 10.5%; 2: 15.8%; 3: 5.3%; 4: 5.3%; 8: 5.3%; 10: 10.5%; 23: 5.3%; 28: 15.8%; 45: 5.3% |
| 1. How many of your patients are being treated with combination chelation and zinc therapy? | Yes: 31.6%; No: 68.4% |
| 1. Approximately how many patients at your center have undergone liver transplant due to complications of Wilson disease? | 0: 42.1%; 1: 10.6%; 2: 26.3%; 3: 5.3%; 4: 10.5%; 8: 5.3% |
| 1. How many patients at your center have passed due to complications from Wilson disease? | 0: 61.9%; 1-3: 33.3%; >3: 4.8% |
| 1. At your center, are patients with Wilson disease followed by a single dedicated specialist (such as a hepatologist)? | Yes: 95.2%; No: 4.8% |
| 1. At your center, are patients with Wilson disease followed by a multidisciplinary team? | Yes: 42.9%; No: 57.1% |
| 1. Does your center perform genetic analysis/screening for Wilson disease? | Yes: 52.4%; No: 47.6% |
| 1. In general, how long does it take to receive the results of genetic analysis/screening? | Within 1 month: 55%; Within 3 months: 30%; Other: 15% |
| 1. Does your center’s laboratory perform 24-hour quantification of urinary copper and zinc? | Yes: 76.2%; No: 23.8% |
| 1. How long does it normally take to receive these results? | Same day: 14.3%; Within 3 days: 14.3%; Within 1 week: 61.9%; Other: 9.5% |
| 1. In the case of a primarily hepatic presentation with no neurologic symptoms, do you always obtain a full neurologic evaluation and/or brain MRI before therapy? | Yes: 71.4%; No: 28.6% |
| 1. Is an eye exam completed for all of your patients to evaluate for Kayser-Fleischer rings? | Yes: 95.2%; No: 0%; In select cases: 4.8% |
| 1. Before starting therapy, do you obtain an ultrasound elastography to stage liver disease? | Yes: 71.4%; No: 14.3%; In select cases: 14.3% |
| 1. Which ultrasound elastography cut off do you use to diagnose cirrhosis in a patient with Wilson disease? | 9.9 kPa: 60%; >12 kPa: 5%; 13 kPa: 20%; Pediatric patient >6 kPa: 5%; No established cutoff: 5%; Not performed: 5% |
| 1. For the diagnosis of Wilson disease, do you obtain a liver biopsy with copper quantification? | Yes: 28.6%; No: 23.8%; In select cases: 47.6% |
| 1. In which case(s) do you think a liver biopsy is indicated? | In staging of liver disease: 38.1%; If other etiologies of liver disease remain on the differential: 71.4%; To confirm the diagnosis if genetic testing is negative: 61.9%; To confirm diagnosis in doubtful cases: 4.6% |
| 1. When you have a diagnosis of Wilson disease, how do you select first line therapy? | As recommended by guidelines: 76.2%; Treatment historically used at my center: 4.8%; As guided by response of other patients at my center: 9.5%; Drug made available from hospital pharmacy: 4.8%; Case by case: 4.8%; Drug with the greatest evidence in literature and clinical practice: 4.8% |
| 1. When you have a patient with a predominantly hepatic presentation, which drug do you use as initial therapy? | d-Penicillamine: 85%; Zinc salt: 10%; Trientine dihydrochloride: 0%; Trientine tetrahydrochloride: 0%; Depends on presence or absence of symptoms: 5% |
| 1. When you have a patient with predominantly neurologic presentation, which drug do you use as initial therapy? | d-Penicillamine: 55%; Zinc salt: 10%; Trientine dihydrochloride: 0%; Trientine tetrahydrochloride: 5%; Chelation therapy + zinc salt: 20%; Depends on cirrhosis and neurologic response to chelators: 5%; Trientine: 5% |
| 1. When you have a patient with a mixed hepatic/neurologic presentation, which drug do you use as initial therapy? | d-Penicillamine: 55%; Zinc salt: 10%; Trientine dihydrochloride: 0%; Trientine tetrahydrochloride: 5%; Chelation therapy + zinc salt: 20%; Depends on cirrhosis and neurologic response to chelators: 5%; Trientine: 5% |
| 1. In case of side effects from d-penicillamine, which second line therapy do you choose? | Zinc salt: 15%; Trientine: 5%; Chelation therapy + zinc: 5%; Trientine dihydrochloride: 15%; Trientine tetrahydrochloride: 55%; Depends on patient’s de-copperization phase: 5% |
| 1. In case of side effects from zinc salt, which second line therapy do you choose? | d-Penicillamine: 40%; Trientine dihydrochloride: 15%; Trientine tetrahydrochloride: 35%; Zinc sulfate/acetate: 5%; Trientine: 5% |
| 1. According to your experience, when do you consider changing from initial therapy to maintenance therapy? | Stable clinical presentation and liver function testing: 4.8%; Stable clinical presentation, liver function testing, and 24 hr urinary copper: 85.7%; There is no difference in initial and maintenance therapy: 9.5% |
| 1. According to your experience, how long does it take for Wilson disease to stabilize before changing to maintenance treatment? | 6 months: 19%; 12 months: 52.4%; 24 months: 14.3%; Depends on urinary copper: 4.7%; Variable: 9.4% |
| 1. In the case of initial treatment with d-penicillamine, which therapy will you use for maintenance? | Same therapy at a lower dose: 61.1%; Same therapy same dose: 0%; Change to zinc salt: 38.9%; Change to trientine: 0% |
| 1. What dose of d-penicillamine do you use as a starting dose? | 17-20mg/kg/day: 45%; 14-16mg/kg/day: 35%; 11-13mg/kg/day: 0%; 9-11mgkg/day: 10%; 10-20 mg/kg/day: 5%; Initial dose 125 mg followed by 250mg every 7 days: 5% |
| 1. What dose of d-penicillamine do you use as maintenance dose? | >15mg/kg/day: 5%; 13-15mg/kg/day: 35%; 11-13mg/kg/day: 25%; 9-11mg/kg/day: 10%; Other: 25% |
| 1. How do you prescribe trientine? | Fixed dose: 0%; Weight based: 85%; By disease severity: 15%; If previous treatment was d-penicillamine, I use that same dosage in mg: 10%; If previous treatment was d-penicillamine, I use a higher dosage in mg: 0% |
| 1. Which starting dose of trientine would you prescribe in a 70 kg patient? | 600-800mg: 12.5%; 800-1000mg: 37.5%; 1000-1200mg: 18.8%; 1200-1400mg: 25%; >1400mg: 6.2% |
| 1. What dosage do you prescribe for maintenance? | I reduce by 20-30%: 68.4%; I reduce by 40%: 5.3%; I do not change dosage for maintenance: 15.8%; Other: 10.5% |
| 1. Which zinc salt do you use? | Zinc sulfate: 5.6%; Zinc acetate: 83.3%; Zinc gluconate: 11.1% |
| 1. During the maintenance of Wilson disease, how often do you order blood tests and copper metabolites? | Every 3 months: 23.8%; Every 6 months: 47.6%; Every year: 19%; Every 2 years: 0%; 6 months to 1 year: 4.3%; Other: 4.8% |
| 1. During the maintenance of Wilson disease, how often do you obtain liver ultrasound? | Every 3 months: 0%; Every 6 months: 47.6%; Every year: 33.3%; Every 2 years: 4.7%; Other: 14.3% |
| 1. During the maintenance of Wilson disease, how often do you obtain ultrasound elastography? | Every 6 months: 9.5%; Every year: 52.4%; Every 2 years: 38.1% |
| 1. In your experience, how many patients are non-compliant with their medications for Wilson disease? | 0-20%: 52.4%; 20-40%: 28.6%; 40-60%: 9.5%; 60-80%: 9.5%: 80-100%: 0% |
| 1. In your experience, how many patients have stopped their medications for Wilson disease of their own accord? | 0-20%: 90.5%; 20-40%: 9.5%; 40-60%: 0%; 60-80%: 0%: 80-100%: 0% |
| 1. During treatment, do you ever recommend repeat liver biopsy with copper quantification to evaluate the progression of liver disease? | Always: 0%; Never: 52.4%; In select cases: 47.6% |
| 1. Once the diagnosis of Wilson disease is established, to which degree do you recommend screening relatives? | First degree: 90.5%; Second degree: 9.5% |
| 1. If you have asymptomatic patients diagnosed during familial screening, which drug will you choose as initial therapy? | D-penicillamine: 9.5%; trientine: 0%; zinc salt: 76.2%; No therapy, monitor: 14.3% |
| 1. Do you change therapy during pregnancy? | I never change therapy during pregnancy: 11.1%; I stop therapy: 0%; I stop chelators and change to zinc salt: 22.2%; I reduce the dose of penicillamine: 61.1%; I reduce the dose of trientine: 5.6% |

**Supplemental Table 2.**

| **Survey question** | **California respondent results** |
| --- | --- |
| 1. What type of patients do you follow? | Adult: 98.2%; Pediatric: 1.7% |
| 1. Do you practice within a referral center for rare disease? | Yes: 48.3%; No: 51.7% |
| 1. Approximately how many adult patients with Wilson disease are followed in your center? | Variable |
| 1. How many patients have you diagnosed with Wilson disease in your professional life? | 0 patients: 29.3%; 1-5: 60.3%; 6-10: 6.9%; 11-20: 1.7%; over 20: 1.7% |
| 1. How many patients have been diagnosed with Wilson disease in the past three years? | 0 patients: 44.8%; 1-5: 71.7%; over 5: 3.4% |
| 1. How many of your patients are being treated with d-penicillamine therapy? | 0 patients: 67.6%; 1: 23.5%; 2: 5.8%; 3: 2.9% |
| 1. How many of your patients are being treated with trientine therapy? | 0 patients: 59.3%; 1: 9.3%; 2: 6.3%; 3: 9.3%; 10: 6.3%; 12: 3.1%; 16: 3.1% |
| 1. How many of your patients are being treated with zinc salts? | 0 patients: 56.2%; 1: 12.5%; 2: 6.3%; 3: 15.6%; 4: 3.1%; 5: 4.1% |
| 1. How many of your patients are being treated with combination chelation and zinc therapy? | Yes: 34.4%; No: 65.6% |
| 1. Approximately how many patients at your center have undergone liver transplant due to complications of Wilson disease? | 0: 56.3%; 1: 15.6%, 2: 6.3%; 3: 9.4%; 5: 9.4%; 10: 3.1% |
| 1. How many patients at your center have passed due to complications from Wilson disease? | 0: 78.9%; 1-3: 13.2%; >3: 7.9% |
| 1. At your center, are patients with Wilson disease followed by a single dedicated specialist (such as a hepatologist)? | Yes: 56.4%; No: 43.6% |
| 1. At your center, are patients with Wilson disease followed by a multidisciplinary team? | Yes: 36.1%; No: 63.9% |
| 1. Does your center perform genetic analysis/screening for Wilson disease? | Yes: 68.4%; No: 31.6% |
| 1. If not, which laboratory does your center utilize? | Variable |
| 1. In general, how long does it take to receive the results of genetic analysis/screening? | Within 1 month: 87.1%; Within 3 months: 0%; Other: 12.9% |
| 1. Does your center’s laboratory perform 24-hour quantification of urinary copper and zinc? | Yes: 91.4%; No: 8.6% |
| 1. How long does it normally take to receive these results? | Same day: 2.9%; Within 3 days: 32.4%; Within 1 week: 55.9%; Other: 8.8% |
| 1. In the case of a primarily hepatic presentation with no neurologic symptoms, do you always obtain a full neurologic evaluation and/or brain MRI before therapy? | Yes: 48.6%; No: 51.4% |
| 1. If yes, do you obtain a neurology consultation or brain MRI? | Neurology consultation: 11.1%; Brain MRI: 44.4%; Both: 44.4% |
| 1. Is an eye exam completed for all of your patients to evaluate for Kayser-Fleischer rings? | Yes: 80.9%; No: 0%; In select cases: 19.1% |
| 1. Before starting therapy, do you obtain an ultrasound elastography to stage liver disease? | Yes: 61.9%; No: 14.3%; In select cases: 23.8% |
| 1. Which ultrasound elastography cut off do you use to diagnose cirrhosis in a patient with Wilson disease? | 9.9 kPa: 23.5%; 14 kPa: 58.8%; Other: 17.6% |
| 1. For the diagnosis of Wilson disease, do you obtain a liver biopsy with copper quantification? | Yes: 50%; No: 10%; In select cases: 40% |
| 1. In which case(s) do you think a liver biopsy is indicated? | In staging of liver disease: 24.1%; If other etiologies of liver disease remain on the differential: 51.7%; To confirm the diagnosis if genetic testing is negative:  24.1% |
| 1. When you have a diagnosis of Wilson disease, how do you select first line therapy? | As recommended by guidelines: 95.2%; Treatment historically used at my center: 0%; As guided by response of other patients at my center: 4.8%; As guided by insurance coverage: 0%; Other: 0% |
| 1. When you have a patient with a predominantly hepatic presentation, which drug do you use as initial therapy? | d-Penicillamine: 26.3%; Zinc salt: 0%; Trientine: 47.4%; Chelation therapy + zinc salt: 10.5%; Other: 15.8% |
| 1. When you have a patient with predominantly neurologic presentation, which drug do you use as initial therapy? | d-Penicillamine: 38.9%; Zinc salt: 16.7%; Trientine: 11.1%; Chelation therapy + zinc salt: 11.1%; Other: 22.2% |
| 1. When you have a patient with a mixed hepatic/neurologic presentation, which drug do you use as initial therapy? | d-Penicillamine: 35.3%; Zinc salt: 5.8%; Trientine: 17.7%; Chelation therapy + zinc salt: 29.4%; Other: 11.8% |
| 1. In case of side effects from d-penicillamine, which second line therapy do you choose? | Zinc salt: 11.8%; Trientine: 70.6%; Chelation therapy + zinc: 11.8%; Other: 5.9% |
| 1. In case of side effects from zinc salt, which second line therapy do you choose? | d-Penicillamine: 11.8%; Trientine: 76.5%; Chelation + zinc: 5.9%; Other: 5.9% |
| 1. According to your experience, when do you consider changing from initial therapy to maintenance therapy? | Stable clinical presentation and liver function testing: 16.7%; Stable clinical presentation, liver function testing, and 24 hr urinary copper: 72.2%; There is no difference in initial and maintenance therapy: 0%; Other: 11.1% |
| 1. According to your experience, how long does it take for Wilson disease to stabilize before changing to maintenance treatment? | Less than 6 months: 18.8%; 6-12 months: 50%; 12-24 months: 12.5%; More than 24 months: 18.8% |
| 1. In the case of initial treatment with d-penicillamine, which therapy will you use for maintenance? | Same therapy at a lower dose: 31.3%; Same therapy same dose: 12.5%; Change to zinc salt: 31.3%; Change to trientine: 18.8%; Other: 6.3% |
| 1. What dose of d-penicillamine do you use as a starting dose? | 17-20mg/kg/day: 28.6%; 14-16mg/kg/day: 35.7%; 11-13mg/kg/day: 0%; 9-11mgkg/day: 14.3%; Other: 21.4% |
| 1. What dose of d-penicillamine do you use as maintenance dose? | >15mg/kg/day: 14.3%; 13-15mg/kg/day: 14.3%; 11-13mg/kg/day: 7.1%; 9-11mg/kg/day: 42.9%; Other: 21.4% |
| 1. How do you prescribe trientine? | Fixed dose: 13.3%; Weight based: 60%; By disease severity: 13.3%; If previous treatment was d-penicillamine, I use that same dosage in mg: 13.3%; If previous treatment was d-penicillamine, I use a higher dosage in mg: 0% |
| 1. Which starting dose of trientine would you prescribe in a 70 kg patient? | 600-800mg: 14.3%; 800-1000mg: 14.3%; 1000-1200mg: 28.6%; 1200-1400mg: 35.7%; >1400mg: 7.1% |
| 1. What dosage do you prescribe for maintenance? | I reduce by 20-30%: 57.1%; I reduce by 40%: 14.3%; I do not change dosage for maintenance: 28.6%; Other: 0% |
| 1. Which zinc salt do you use? | Zinc sulfate: 46.2%; Zinc acetate: 30.8%; Zinc gluconate: 15.4%; Other: 7.7% |
| 1. During the maintenance of Wilson disease, how often do you see patient in clinic for follow-up? | Every 3 months: 26.7%; Every 6 months: 60%; Every year: 6.7%; Every 2 years: 0%; Other: 6.7% |
| 1. During the maintenance of Wilson disease, how often do you order blood tests? | Every 3 months: 26.7%; Every 6 months: 73.3%; Every year: 0%; Every 2 years: 0%; Other: 0% |
| 1. During the maintenance of Wilson disease, how often do you order copper metabolites? | Every 3 months: 13.3%; Every 6 months: 60.0%; Every year: 20.0%; Every two years: 6.7%; Other: 0% |
| 1. During the maintenance of Wilson disease, how often do you obtain liver ultrasound? | Every 3 months: 0%; Every 6 months: 53.3%; Every year: 13.3%; Every 2 years: 26.7%; Other: 6.7% |
| 1. During the maintenance of Wilson disease, how often do you obtain ultrasound elastography? | Every 3 months: 0%; Every 6 months: 13.3%; Every year: 33.3%; Every 2 years: 46.7%; Other: 6.7% |
| 1. In your experience, how many patients are non-compliant with their medications for Wilson disease? | 0-20%: 31.3%; 20-40%: 50%; 40-60%: 18.8%; 60-80%: 0%: 80-100%: 0% |
| 1. In your experience, how many patients have stopped their medications for Wilson disease of their own accord? | 0-20%: 68.8%; 20-40%: 25%; 40-60%: 6.3%; 60-80%: 0%: 80-100%: 0% |
| 1. During treatment, do you ever recommend repeat liver biopsy with copper quantification to evaluate the progression of liver disease? | Always: 0%; Never: 13.3%; In select cases: 86.7% |
| 1. Once the diagnosis of Wilson disease is established, to which degree do you recommend screening relatives? | First degree: 100%; Second degree: 0%; Other: 0% |
| 1. If you have asymptomatic patients diagnosed during familial screening, which drug will you choose as initial therapy? | D-penicillamine: 13.3%; trientine: 6.7%; zinc salt: 53.3%; No therapy, monitor: 26.7%; Other: 0% |
| 1. Do you change therapy during pregnancy? | I never change therapy during pregnancy: 6.3%; I always change therapy during pregnancy: 25%; In select cases, I change therapy: 68.8% |

**Appendix 1. California Survey questions**

1. What type of patients do you follow?
   1. Adult
   2. Pediatric
2. Do you practice within a referral center for rare disease?
   1. Yes
   2. No
3. Approximately how many adult patients with Wilson disease are followed in your center?
4. How many patients have you diagnosed with Wilson disease in your professional life?
5. How many patients have been diagnosed with Wilson disease in the past three years?
6. How many of your patients are being treated with d-penicillamine therapy?
7. How many of your patients are being treated with trientine therapy?
8. How many of your patients are being treated with zinc salts?
9. How many of your patients are being treated with combination chelation and zinc therapy?
10. Approximately how many patients at your center have undergone liver transplant due to complications of Wilson disease?
11. How many patients at your center have passed due to complications from Wilson disease?
12. 0
13. 1-3
14. >3
15. At your center, are patients with Wilson disease followed by a single dedicated specialist (such as a hepatologist)?
16. Yes
17. No
18. At your center, are patients with Wilson disease followed by a multidisciplinary team?
19. Yes
20. No
21. Does your center perform genetic analysis/screening for Wilson disease?
22. Yes
23. No
24. If not, which laboratory does your center utilize?
25. In general, how long does it take to receive the results of genetic analysis/screening?
26. Within 30 days
27. Within 1 month
28. Within 3 months
29. Other
30. Does your center’s laboratory perform 24-hour quantification of urinary copper and zinc?
31. Yes
32. No
33. How long does it normally take to receive these results?
34. Same day
35. Within 3 days
36. Within one week
37. Other
38. In the case of a primarily hepatic presentation with no neurologic symptoms, do you always obtain a full neurologic evaluation and/or brain MRI before therapy?
39. Yes
40. No
41. If yes, do you obtain a neurology consultation or brain MRI?
42. Neurology consult
43. Brain MRI
44. Both
45. Is an eye exam completed for all of your patients to evaluate for Kayser-Fleischer rings?
46. Yes
47. No
48. In select cases
49. Before starting therapy, do you obtain an ultrasound elastography to stage liver disease?
50. Yes
51. No
52. In select cases
53. Which ultrasound elastography cut off do you use to diagnose cirrhosis in a patient with Wilson disease?
54. 9.9 kPa
55. 14 kPa
56. Other
57. For the diagnosis of Wilson disease, do you obtain a liver biopsy with copper quantification?
58. Yes
59. No
60. In select cases
61. In which case(s) do you think a liver biopsy is indicated?
62. In the staging of liver diseae
63. If other etiologies of liver disease remain on the differential
64. To confirm the diagnosis if genetic testing is negative
65. Other
66. When you have a diagnosis of Wilson disease, how do you select first line therapy?
67. As recommended by guidelines
68. Treatment historically used in my center
69. As guided by the response of other patients followed at my center
70. As guided by insurance coverage
71. Other
72. When you have a patient with a predominantly hepatic presentation, which drug do you use as initial therapy?
73. D-penicillamine
74. Zinc salt
75. Trientine
76. Chelation therapy and zinc salt
77. Other
78. When you have a patient with predominantly neurologic presentation, which drug do you use as initial therapy?
79. D-penicillamine
80. Zinc salt
81. Trientine
82. Chelation therapy and zinc salt
83. Other
84. When you have a patient with a mixed hepatic/neurologic presentation, which drug do you use as initial therapy?
85. D-penicillamine
86. Zinc salt
87. Trientine
88. Chelation therapy and zinc salt
89. Other
90. In case of side effects from d-penicillamine, which second line therapy do you choose?
91. Zinc salt
92. Trientine
93. Chelation therapy and zinc salt
94. Other
95. In case of side effects from zinc salt, which second line therapy do you choose?
96. D-penicillamine
97. Trientine
98. Chelation therapy and zinc salt
99. Other
100. According to your experience, when do you consider changing from initial therapy to maintenance therapy?
101. Stable clinical presentation and liver function testing
102. Stable clinical presentation, liver function testing and 24-hour urinary copper
103. There is no difference in initial and maintenance therapy
104. Other
105. According to your experience, how long does it take for Wilson disease to stabilize before changing to maintenance treatment?
106. Less than 6 months
107. 6-12 months
108. 12-24 months
109. More than 24 months
110. In the case of initial treatment with d-penicillamine, which therapy will you use for maintenance?
111. The same therapy at a lower dose
112. The same therapy at the same dose
113. Change to zinc salt
114. Change to trientine
115. Other
116. What dose of d-penicillamine do you use as a starting dose?
117. 17-20 mg/kg/day
118. 14-16 mg/kg/day
119. 11-13 mg/kg/day
120. 9-11 mg/kg/day
121. Other
122. What dose of d-penicillamine do you use as maintenance dose?
123. > 15 mg/kg/day
124. 13-15 mg/kg/day
125. 11-13 mg/kg/day
126. 9-11 mg/kg/day
127. How do you prescribe trientine?
128. Fixed dose
129. Weight based
130. By disease severity
131. If the previous treatment was d-penicillamine, I use that same dosage in mg
132. If the previous treatment was d-penicillamine, I use a higher dosage in mg
133. Which starting dose of trientine would you prescribe in a 70 kg patient?
134. 600-800 mg
135. 800-1000 mg
136. 1000-1200 mg
137. 1200-1400 mg
138. >1400 mg
139. What dosage do you prescribe for maintenance?
140. I reduce by 20-30%
141. I reduce by 40%
142. I do not change dosage for maintenance
143. Other
144. Which zinc salt do you use?
145. Zinc sulfate
146. Zinc acetate
147. Zinc gluconate
148. Other
149. During the maintenance of Wilson disease, how often do you see patient in clinic for follow-up?
150. Every 3 months
151. Every 6 months
152. Every year
153. Every two years
154. Other
155. During the maintenance of Wilson disease, how often do you order blood tests?
156. Every 3 months
157. Every 6 months
158. Every year
159. Every two years
160. Other
161. During the maintenance of Wilson disease, how often do you order copper metabolites?
162. Every 3 months
163. Every 6 months
164. Every year
165. Every two years
166. Other
167. During the maintenance of Wilson disease, how often do you obtain liver ultrasound?
168. Every 3 months
169. Every 6 months
170. Every year
171. Every two years
172. Other
173. During the maintenance of Wilson disease, how often do you obtain ultrasound elastography?
174. Every 3 months
175. Every 6 months
176. Every year
177. Every two years
178. Other
179. In your experience, how many patients are non-compliant with their medications for Wilson disease?
180. 0-20%
181. 20-40%
182. 40-60%
183. 60-80%
184. 80-100%
185. In your experience, how many patients have stopped their medications for Wilson disease of their own accord?
186. 0-20%
187. 20-40%
188. 40-60%
189. 60-80%
190. 80-100%
191. During treatment, do you ever recommend repeat liver biopsy with copper quantification to evaluate the progression of liver disease?
192. Always
193. Never
194. In select cases
195. Once the diagnosis of Wilson disease is established, to which degree do you recommend screening relatives?
196. 1^st^
197. 2^nd^
198. Other
199. If you have asymptomatic patients diagnosed during familial screening, which drug will you choose as initial therapy?
200. D-penicillamine
201. Trientine
202. Zinc salt
203. No therapy, solely monitor
204. Other
205. Do you change therapy during pregnancy?
206. I never change therapy during pregnancy
207. I always change therapy during pregnancy
208. In select cases, I change therapy during pregnancy

**Supplemental figure. Neurologic evaluation after initiation of therapy in hepatic presentation**
